# Molecularly Distinct NLRP3 Inducers Mediate Diverse Ratios of Interleukin-1*β* and Interleukin-18 from Human Monocytes

**DOI:** 10.1155/2020/4651090

**Published:** 2020-10-22

**Authors:** Kristine Midtbö, Daniel Eklund, Eva Särndahl, Alexander Persson

**Affiliations:** ^1^School of Medical Sciences, Faculty of Medicine and Health, Örebro University, SE-701 82 Örebro, Sweden; ^2^Inflammatory Response and Infection Susceptibility Centre (iRiSC), Faculty of Medicine and Health, Örebro University, SE-701 82 Örebro, Sweden

## Abstract

Inflammasomes cleave and activate interleukin- (IL-) 1*β* and IL-18 which have both shared and unique biological functions. IL-1*β* is an important mediator of the acute phase response to infections and tissue damage, whereas IL-18 takes part in activation and tailoring of the adaptive immune response. While IL-1*β* has served as the prototypic indicator of inflammasome activation, few studies have compared the potential differences in IL-1*β* and IL-18 production during inflammasome activation. Since these cytokines partake in different immune pathways, the involvement of inflammasome activity in different conditions needs to be described beyond IL-1*β* production alone. To address a potential heterogeneity in inflammasome functionality, ATP, chitosan, or silica oxide (SiO_2_) were used to induce NLRP3 inflammasome activation in THP-1 cells and the subsequent outcomes were quantified. Despite using doses of the inflammasome inducers yielding similar release of IL-1*β*, SiO_2_-stimulated cells showed a lower concentration of released IL-18 compared to ATP and chitosan. Hence, the cells stimulated with SiO_2_ responded with a distinctly different IL-18 : IL-1*β* ratio. The difference in the IL-18 : IL-1*β* ratio for SiO_2_ was constant over different doses. While all downstream responses were strictly dependent on a functional NLRP3 inflammasome, the differences did not depend on the level of gene expression, caspase-1 activity, or pyroptosis. We suggest that the NLRP3 inflammasome response should be considered a dynamic process, which can be described by taking the ratio between IL-1*β* and IL-18 into account and moving away from an on/off perspective of inflammasome activation.

## 1. Introduction

IL-1*β* and IL-18 are crucial for mounting a potent proinflammatory response and directing subsequent immune responses. Activation of interleukin- (IL-) 1*β* and IL-18 into their biologically active forms require the formation of a multiprotein complex called inflammasome [1]. Today, several inflammasomes have been described, with the nomenclature depending on the main Nod-like receptor (NLR) involved. Upon activation, the NLRs oligomerize to form the core of the inflammasome complex by recruiting the adaptor protein apoptosis-associated speck-like protein containing a CARD (ASC). ASC in turn recruits and activates the serine protease caspase-1 that is the effector caspase responsible for the bioactivation of IL-1*β*, IL-18, and gasdermin D (GSDMD) [2, 3].

Due to the inflammatory potency in the cytokines regulated by inflammasomes, activation of the inflammasome is under strict regulation and requires two distinct signals. Signal one upregulates inflammasome components and the pro-form of IL-1*β* [3], while the second signal can then activate a specific NLR, initiating the formation of the inflammasome complex. Whereas many NLRs such as AIM2 and NLRC4 initiate inflammasome formation as a response to specific agonists, the NLRP3 inflammasome can be activated by a broad range of stimuli related to both pathogens, cellular stress and tissue damage. The NLRP3 inflammasome is by far the most studied inflammasome, and the vast array of potential inducers renders it unlikely that the NLRP3 receptor directly interacts with the agonists [4, 5]. Among the characterized inflammasome inducers are adenosine triphosphate (ATP), a nucleotide and well-known danger signal which activates NLRP3 through K^+^ efflux after binding to the ATP receptor P2X7 [6–9]. Chitosan, a family of linear polysaccharides found in fungal cell walls, is believed to activate the NLRP3 by lysosomal disruption [10], K^+^ efflux, and/or through induction of reactive oxygen species [11, 12]. Inorganic silica oxide crystals (SiO_2_) have been reported to activate the NLRP3 inflammasome through lysosome disruption, induction of mitochondrial damage [13, 14], or alternatively through the scavenger receptor SR-B1 [15].

Production of IL-1*β* is often used as the proxy for describing inflammasome activation. However, using this benchmark indicates that inflammasome activation is a static mechanism, and the possibility of different signaling pathways inducing different effects on the functional aspects following inflammasome activation are generally not discussed. Active IL-1*β* and IL-18 share several functions as proinflammatory cytokines, but they also have unique properties resulting in distinct immunological profiles. IL-1*β* is a pyrogen that induces fever, which is a property that IL-18 lack [16, 17]. Other features of IL-1*β* include neutrophil mobilization, acute phase protein production, Th17 differentiation, and enhanced antigen presentation and glycolytic rate in Th17 cells and macrophages [16–20]. On the other hand, IL-18 promotes Th1 or Th2 differentiation, depending on the cytokine environment [21], and induces IFN-*γ* production from Th1 cells and NK-cells as well as IL-13 and IL-4 from basophils and mast cells [22].

IL-1*β* and IL-18 also contribute to the pathogenesis of many diseases, but they play different roles. Since IL-1*β* promotes Th17 differentiation, IL-1*β* can be related to many Th17-driven diseases, including rheumatoid arthritis, multiple sclerosis, and psoriasis [23–25]. IL-1*β* also take part in the pathogenesis of gout, osteoarthritis, inflammatory bowel disease, pericarditis, macrophage activating syndrome, chronic obstructive pulmonary disease (COPD) [26–31], and chronic systemic inflammatory conditions, like cryopyrin-associated periodic syndrome (CAPS), adult-onset Still's disease, and familial Mediterranean fever (FMF) [26, 32, 33]. On the other hand, IL-18 is strongly related to IFN-*γ* and Th1/Th2 diseases, like systemic lupus erythematosus, Crohn's disease, and graft versus host rejection [16, 31, 34, 35]. IL-18 is also a key player in allergy, atherosclerosis, dermatitis, acute renal ischemia, hepatitis, and heart failure [31, 36–41]. However, even if IL-1*β* or IL-18 may play a dominant role, both often contribute to the disease but play separate roles at different stages of diseases. Knockout studies in a mouse model of CAPS showed that loss of the IL-1*β* or IL-18 receptor was differently beneficial in young and old mice [42]. Additionally, FMF have shown to be correlated to elevated levels of IL-18, while IL-1*β* play a dominant role during inflammatory flare ups that are treated with IL-1*β* inhibition [43, 44]. Several approved therapies target the IL-1 receptor (IL-1R) or free circulating IL-1*β*, including the IL-1R antagonist anakinra (Kineret®). This have been proven to be successful in many cases but since targeting IL-1*β* does not inhibit the effect of IL-18, it is not surprising that certain conditions are unaffected by the treatment [45], and recently, efforts have been made to investigate the effectiveness of instead blocking IL-18 in certain inflammatory diseases [41, 46, 47]. Since IL-1*β* and IL-18 contribute diversely to different diseases, it is unlikely that inflammasome activation is a static on/off process. Consequently, when studying inflammasome functionality, the activity may need to be described beyond IL-1*β* production. This study was therefore designed to describe the outcome of NLRP3 inflammasome activation and the functional effects of diverse inflammasome inducers.

## 2. Materials and Methods

### 2.1. Cell Culture

THP-1 cells were cultured in RPMI medium 1640 (Gibco, Thermo Fisher Scientific, Waltham, MA cat. nr:31870-025) supplemented with 10% fetal bovine serum (FBS), Hepes (10 mM), sodium pyruvate (1 mM), GlutaMax (2 mM), glucose (2.5 g/L), and penicillin-streptomycin (PEST, 100 U/mL) all from Gibco (Thermo Fisher Scientific). The THP-1 cell lines used are THP-1 null cells (thp-null), THP-1 defNLRP3 cells (rhp-dnlp), THP-1 defASC cells (thp-dasc), THP-1 defCASP1 (thp-dcasp1), and THP-1 Xblue™-MD2-CD14 cells (thp-mdcdsp) which were all acquired from InvivoGen (San Diego, CA). The THP-1 cells were kept undifferentiated as a model of human monocytes.

### 2.2. Isolation of Primary Human Monocytes

Human CD14^+^ cells were isolated from healthy volunteers. PBMCs were separated by density gradient centrifugation on Lymphoprep (Axis-Shield, Oslo, Norway), and the CD14^+^ cells were isolated by magnetic sorting using CD14 MACS microbeads (Miltenyi Biotech, Bergisch Gladbach, Germany) according to the manufacturer's protocol. The cells were cultured in DMEM medium (Lonza BioWhittaker, Thermo Fisher Scientific cat. nr: 11635220) supplemented with 10% human AB serum (pooled from five healthy volunteers), L-glutamine (5 mM), sodium pyruvate (5 mM), and glucose (2.5 g/L) (Gibco, Thermo Fisher Scientific).

### 2.3. Experimental Setup

**Figure 1:**
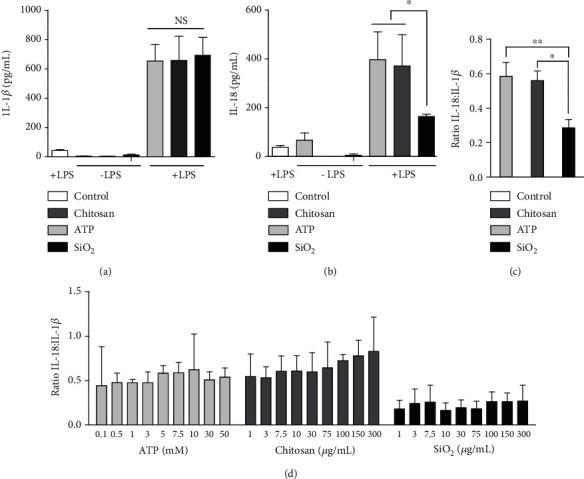
ATP, chitosan, and SiO_2_ induce diverse IL-18 : IL-1*β* ratio in THP-1 cells. The concentration of released IL-1*β* (a) and IL-18 (b) in nonprimed (-LPS) and LPS-primed (+LPS, 100 ng/mL) cells, 24 h post stimulation were detected after stimulation with 5 mM ATP, 30 *μ*g/mL chitosan, or 30 *μ*g/mL SiO2. The ratio between IL-1*β* and IL-18 was calculated for each experiment (c) and after stimulation with increasing doses of ATP (mM), chitosan (*μ*g/mL), or SiO_2_ (*μ*g/mL) (d). Controls were left untreated (-LPS) or treated with LPS alone (+LPS). Data are shown as mean ± SD from three-six individual experiments. NS: non significant, ^∗^*p* < 0.05; ^∗∗^*p* < 0.01.

THP-1 cells were seeded into 96- or 24-well plates and primed with lipopolysaccharide- (LPS-) B5 (E. coli serotype 055:K59(B5)H-, 100 ng/mL, or concentrations indicated in Figure 1(d)) for 10 minutes to induce instant priming, followed by additional stimulation with ATP (5 mM), chitosan (30 *μ*g/mL), or SiO_2_ (30 *μ*g/mL) all from InvivoGen, for 24 h.

Isolated primary human monocytes were primed with 10 ng/mL LPS prior to stimulation with 10 nM ATP, 10 ng/mL chitosan, or 10 ng/mL SiO_2._

### 2.4. Cytokine Measurement

The concentrations of cytokines were measured with enzyme-linked immunosorbent assay (ELISA) for IL-1*β* (BioLegend, San Diego, CA) and IL-18 (R&D Systems, Minneapolis, MN) according to the manufacturer's instruction.

### 2.5. SEAP Reporter Assay

NF-*κ*B activity was measured indirectly by the SEAP activity from a NF-*κ*B responsive SEAP reporter gene in the reporter THP-1 xBlue™ cells. The accumulation of SEAP in the culture medium 24 h after stimulation was detected with the QUANTI-Blue™ (InvivoGen) according to the manufacturer's instructions. The OD was measured at 635 nm with Cytation 3 imaging plate reader (BioTek, Winooski, VT).

### 2.6. FLICA Assay

Activation of caspase-1 was detected with the fluorescent probe FAM-YVAD-FMK (FLICA) from Immunochemistry Technologies (Bloomington, MN cat.nr: OKSA11275), that binds irreversibly to active caspase-1. The cells were stained for 1 h and washed two times in PBS prior to analysis. The percentage of positive cells was measured by flow cytometry, (Accuri™ C6, BD, Franklin Lakes, NJ).

### 2.7. Lactate Dehydrogenase Assay

Cellular rupture was detected using the Pierce® lactate dehydrogenase (LDH) cytotoxicity assay kit (Thermo Scientific) according to the manufacturer's instructions. Data are presented as percentage LDH calculated from a positive control (100% lysed cells).

### 2.8. Western Blotting

Cells used for Western blotting were stimulated in FBS-free media. Cells were lysed in RIPA lysis buffer (Merck Millipore, Burlington, MA), and the protein concentrations were measured using the Micro BCA™ Protein Assay (Thermo Scientific). The proteins were separated in 8-16% stain-free TGX gels (Bio-Rad, Hercules, CA), transferred to PVDF membranes (iBlot® 2 PVDF regular stacks, Invitrogen, Carlsbad, CA), and analysed by immunoblotting. The primary antibodies used were cleaved IL-1*β* (1 : 1000 Cell Signaling Technologies, Danvers, MA. cat.nr:12242), IL-18 (1 : 1000 Abcam, Cambridge, UK. cat.nr: EPR19954-188), and caspase-1 p20 (1 : 750 Adipogen Life Sciences, cat.nr:AG-20B-0048-C100). Secondary antibodies used were goat anti mouse (1 : 5000 Abcam cat.nr: Ab6789), rabbit anti goat (1 : 2000 Dako, Agilent. Santa Clara, CA. cat.nr: P0160), and goat anti-rabbit (1 : 3000 Invitrogen cat.nr: A11034). Membranes were washed in TBST buffer and analysed with ChemiDoc™ MP Imaging System (Bio-Rad).

### 2.9. Extraction of mRNA, Reverse Transcription, and qPCR

Cells were lysed in RLT lysis buffer (Qiagen, Hilden, Germany) and drawn through a 22 G needle to homogenize the sample. Total RNA was extracted and purified with the QIAmp RNeasy Mini kit (Qiagen). RNA was quantified using NanoDrop 2000 (Thermo Fisher Scientific), and a High-Capacity cDNA Transcription kit (Thermo Fisher Scientific) was used for the reverse transcription reactions (900 ng of total RNA per 60 *μ*L reaction) on a LifePro Thermal Cycler (Bioer, Hangzhou, China). Quantitative real-time PCR was performed using TaqMan assays in QuantStudio 7 Flex PCR (Applied Biosystems, Thermo Fisher Scientific). The TaqMan assays used were *IL1B* (Hs01555410), *IL18* (Hs01038788_m1), *NLRP3* (Hs00918082_m1), *ASC/PYCARD* (Hs01547324_gH), *CASP1* (Hs00354836_m1), *CASP8* (Hs01018151_m1), *HPRT1* (Hs02800695_m1), and *TBP* (Hs00427620_m1), (all from Thermo Fisher Scientific). A comparative quantification was used, where the quantity of each experimental sample was determined using a standard curve as calibrator samples. Calibrator was prepared from human peripheral blood mononuclear cell (PBMC) cultures stimulated for 48 h with 1 *μ*g/mL LPS known to express the genes of interest in high abundance. A six-point serially fourfold diluted standard curve was developed from the calibrator by plotting the threshold cycles versus the dilution factor and the data fitted to a straight line, while confirming that the correlation coefficient (R2) for the line was 0.99 or greater. This plot was then used for extrapolating relative expression level information for the same gene of interest in unknown experimental samples. The relative quantity for the gene of interest was normalized to that of a reference gene in the same sample, and then, the normalized numbers was compared between samples. The reference genes used for normalization, *HPRT1* and *TBP*, were selected from four candidate genes using NormFinder R package (MOMA, Aarhus University Hospital, Denmark), where the geometric mean of said genes were used. RNA from stimulated PBMCs was extracted using QIAamp RNA Blood Mini Kit (Qiagen, Hilden, Germany) and converted into cDNA using the above protocols. 384-well plates were prepared using a PIRO Pipetting Robot (Dornier, Lindau, Germany). Cycle threshold (CT) cut-off value was set to 35 cycles. An acceptable coefficient of variation (CV) between duplicates was set to <15%. Water was used as the negative control.

### 2.10. Statistics

Statistical analyses were performed using GraphPad Prism 5. *p* values were assessed using two-tailed Student's *t*-tests and two-way analysis of variance (ANOVA) followed by Bonferroni's posttest. In the figures, ^∗^*p* < 0.05, ^∗∗^*p* < 0.01, and ^∗∗∗^*p* < 0.001. All data shown are mean ± SD for a minimum of three independent experiments.

## 3. Results

### 3.1. IL-1*β* and IL-18 Release Is Mediated by Inflammasome Inducers ATP, Chitosan, or SiO_2_

To confirm NLRP3 inflammasome activation by ATP, chitosan, and SiO_2_, respectively, and their dependency on a priming signal, cells were primed with two doses of LPS. Cells stimulated with 1 *μ*g/mL LPS alone showed elevated levels of IL-1*β*, uncharacteristic of a true priming signal and only minor changes in IL-1*β* and IL-18 production upon stimulation with ATP, chitosan, or SiO_2_. However, 100 ng/mL LPS induced only low concentrations of IL-1*β* and led to significant synergistic effect upon addition of inflammasome inducers (supplementary figure 1). Henceforth, all experiments performed on THP-1 cells were primed with 100 ng/mL LPS. To investigate potential differences in the response following inflammasome activation, concentrations of the inducers were titrated to doses resulting in similar IL-1*β* levels released 24 h after stimulation (supplementary Figure 2. Stimulation of primed cells with 5 mM ATP, 30 *μ*g/mL chitosan, or 30 *μ*g/mL SiO_2_ induced a significantly increased amount of IL-1*β* compared to primed controls but showed no significant difference in the concentration of released IL-1*β* when compared to each other (Figure 1(a)). However, stimulation with SiO_2_ generated significantly lower levels of IL-18 compared to ATP and chitosan (Figure 1(b)). In terms of ratios, the IL-18 : IL-1*β* ratio for ATP and chitosan was 1 : 0.6 in contrast to 1 : 0.2 for SiO_2_ (Figure 1(c)). The divergent ratio of SiO_2_ remained constant over a broad range of doses, indicating that the ratio is not sensitive to the amount of inducer (Figure 1(d)).

### 3.2. Gene Expression of Inflammasome Components in ATP-, Chitosan-, or SiO_2_-Stimulated Cells

**Figure 2:**
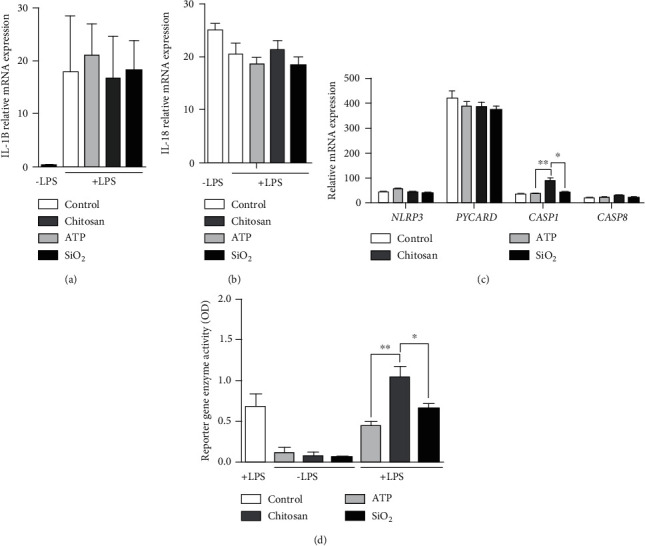
Gene expression of inflammasome components and cytokines do not correlate with the IL-18 : IL-1*β* ratio. The relative mRNA expression of IL1B (a), IL18 (b), and the NLRP3 inflammasome components (c) NLRP3, ASC, and CASP1 as well as CASP8 were measured 24 h after cells primed with LPS were stimulated with ATP, chitosan, or SiO_2_ using qPCR. NF-*κ*B activation was detected indirectly by measuring the activation of the reporter gene SEAP (d). Controls were treated with LPS alone. Data are shown as mean ± SD from six individual experiments. ^∗^*p* < 0.05; ^∗∗^*p* < 0.01.

To elucidate whether the differences in IL-18 : IL-1*β* ratio could be explained by changes in expression levels of inflammasome components and cytokines, gene expression levels *of IL1B, IL18, NLRP3, PYCARD* and *CASP1* were analysed. None of the inducers showed any effect on *IL1B* and *IL18* mRNA expression in LPS-primed cells (Figures 2(a) and 2(b)). Chitosan alone increased the *CASP1* expression (Figure 2(c)). No significant change was seen for *NLRP3* or the potential noncanonical route of activation through *CASP8*. Furthermore, the inducers showed no own effect on NF-*κ*B activation in unprimed cells. However, chitosan showed a synergistic increase in LPS-primed cells, as measured by utilizing the reporter gene SEAP in a modified THP-1 cell line (Figure 2(d)).

### 3.3. Activation of Caspase-1 and Induction of Cell Lysis by Diverse Inducers

**Figure 3:**
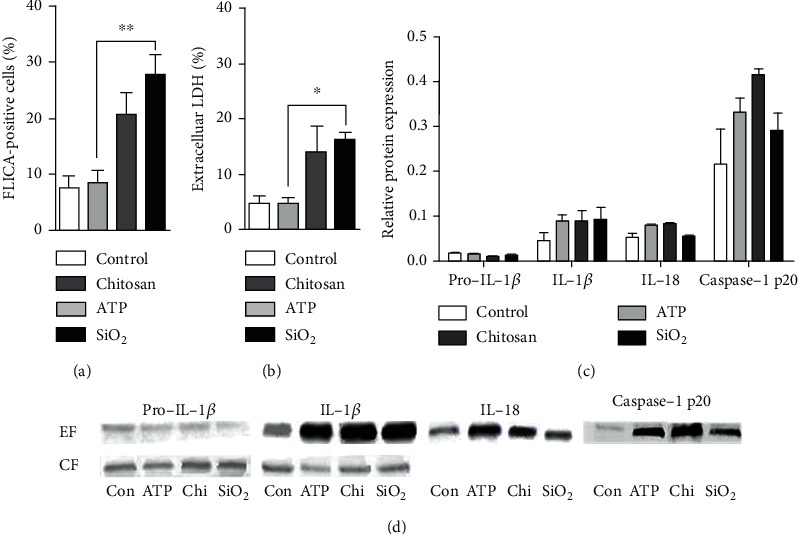
Caspase-1 activity and cell lysis do not correlate with the IL-1*β* : IL-18 ratio. Caspase-1 activity was measured by flow cytometry using the FLICA probe (a), while LDH assay was used as a measure of cell rupture (b). Pro-IL-1*β*, cleaved IL-1*β*, IL-18, and caspase-1 p20 were detected in the extracellular fraction (EF) or cellular lysate as the cellular fraction (CF) by Western blot. The blot density was normalized against the total protein load to calculate the fraction (c). Representative blots are shown in (d). Measurement took place 24 h after stimulation, and controls were treated with LPS alone. Data are shown as mean ± SD from six individual experiments. ^∗^*p* < 0.05; ^∗∗^*p* < 0.01.

To further investigate if observed differences in the IL-18 : IL-1*β* ratio could be attributed to differences in caspase-1 activation, FLICA staining was used to quantify caspase-1 activity, and the presence of pro- and cleaved caspase-1 was detected by Western blot. Chitosan and SiO_2_ but not ATP resulted in an increased percentage of FLICA-positive cells compared to primed controls, 24 h after stimulation (Figure 3(a)). No difference in the protein expression of pro-caspase-1 could be seen compared to the control for any of the inducers (data not shown), and any tendency of differentiating the expression of pro-IL-1*β* and cleaved caspase-1 p20 fragment was statistically nonsignificant (Figures 3(c) and 3(d)). Furthermore, an LDH assay was used to examine the percentage of cell lysis 24 h after stimulation with the inducers. Chitosan and SiO_2_ stimulation resulted in increased LDH release compared to the primed control (Figure 3(b)). The only significant difference between the inducers with regard to both LDH release and FLICA staining was found between ATP and SiO_2_. The LDH results were validated by annexin V and 7AAD staining detected by flow cytometry (data not shown).

### 3.4. Release of Cytokines in Inflammasome-Deficient Cells by Diverse Inducers

**Figure 4:**
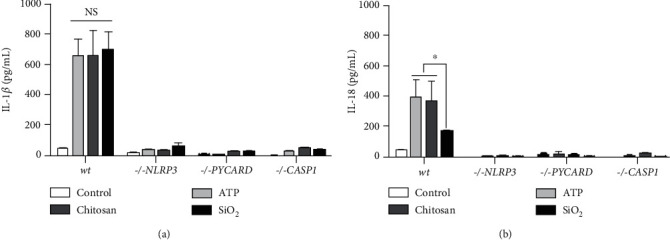
Release of IL-1*β* and IL-18, induced by ATP, chitosan, or SiO_2_ is strictly NLRP3 inflammasome dependent. The concentrations of released IL-1*β* (a) and IL-18 (b) were measured from LPS-primed THP-1 monocytes with functional NLRP3 inflammasome (wt) and inflammasome-deficient THP-1 cells, lacking either functional NLRP3 (-/-NLRP3), ASC (-/-PYCARD), or caspase-1 (-/- CASP1) following stimulation with ATP, chitosan, or SiO_2_. Controls were treated with LPS alone. Data are shown as mean ± SD from six individual experiments. NS: nonsignificant. ^∗^*p* < 0.05.

The relevance of the NLRP3 inflammasome components for the response to the inducers were investigated using THP-1 knockout cells, lacking functional NLRP3, ASC, or caspase-1. Absence of NLRP3, ASC, and caspase-1, respectively, completely attenuated the release of IL-1*β* and IL-18 (Figures 4(a) and 4(b)). While the inducers stimulated the release of other cytokines, not directly regulated by the inflammasome, (supplementary table I), this release was attenuated in inflammasome-deficient cells for several of the cytokines (supplementary figure 3), showing a primary dependency on inflammasome activation for subsequent responses.

### 3.5. IL-18 : Il-1*β* Ratios in Primary Monocytes

**Figure 5:**
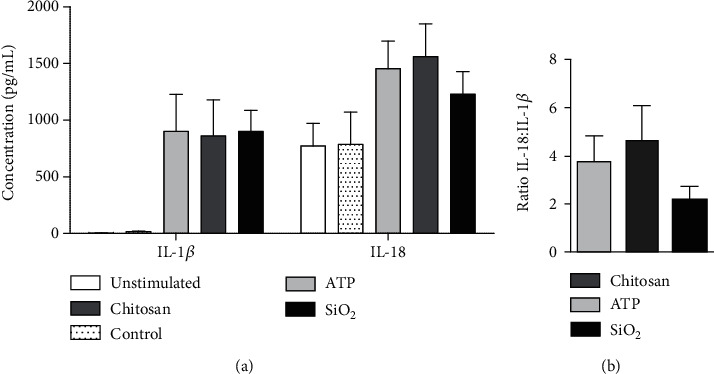
ATP, chitosan, and SiO_2_ induce release of IL-1*β* and IL-18 in primary monocytes. Primary human monocytes were isolated from peripheral blood from healthy volunteers. The release of IL-1*β* and IL-18 was measured after stimulation with 10 nM ATP, 10 ng/mL chitosan, or 10 ng/mL SiO_2_ from LPS-primed cells (10 ng/mL) (a). The IL-18 : IL-1*β* ratios were calculated (b). Data are shown as mean ± SD from six individual experiments.

Lastly, in order to investigate whether the results found in the THP-1 cell line is reflected in primary human cells, monocytes were isolated from healthy volunteers, primed with LPS and stimulated with ATP, chitosan, or SiO_2_. As with the THP-1 cells, the doses of inducers were titrated to achieve the same concentration of released IL-1*β* (supplementary Figure 2). However, a lower dose of LPS was required for priming primary human monocytes compared to THP-1 cells. At this dose, a synergistic effect by the inflammasome inducers was observed without risking the activation of the noncanonical pathway of inflammasome activation as previously reported by Gaidt et al. [48]. However, unlike the THP-1 cells, the concentrations of released IL-18 were higher than for IL-1*β* for all inducers. Although not significant, monocytes responded with a similar pattern, as seen in THP-1 cells, with regard to IL-18 : IL-1*β* ratios (Figures 5(a) and 5(b)).

## 4. Discussion

NLRP3 inflammasome can be activated by numerous inducers and several pathways leading to inflammasome activation have been described. In this study, the differences in functional outcome of three diverse inflammasome inducers, ATP, chitosan, and SiO_2_, with respect to their ability to activate the inflammasome to produce IL-1*β* and IL-18 were investigated. Despite inducing the same level of IL-1*β* release, the studied inducers showed a diverse ability to induce the production of IL-18. This diverse IL-18 : IL-1*β* ratio remained throughout a broad span of doses. While all three inducers showed a strict dependency on priming with LPS and a functional NLRP3 inflammasome, differences could not be attributed to caspase-1 activity, induction of gene expression, or their ability to induce cell death. Furthermore, these inducers exerted diverse effects on other inflammatory cytokines in an NLRP3-dependent fashion. This strengthens the idea that inflammasome activity is highly context-dependent and flexible process that cannot be regarded as an on/off process.

In this study, SiO_2_ stimulation resulted in a lower release of IL-18 compared to ATP and chitosan, which also resulted in a different IL-18 : IL-1*β* ratio. Since IL-1*β* and IL-18 have both shared and unique biological properties, the ratio between them may impact the overall immunological profile, including Th1 or Th17 differentiation [16]. The inducers used in this study represent the broad range of different stimuli that the NLRP3 inflammasome can respond to ATP is a DAMP involved in autoinflammatory diseases that arise during sterile inflammatory conditions, including CAPS and FMF, conditions involving both IL-1*β* and IL-18 [49, 50]. Meanwhile, PAMP-induced inflammation may result in Th1, Th2, or Th17 differentiation, depending on the type of pathogen. Fungal chitosan is a PAMP, and clearance of fungi requires activation of Th1 and Th17 and therefore relies on both IL-1*β* and IL-18 for efficient clearance [16, 51, 52]. SiO_2_ on the other hand induces inflammation as a result of environmental exposure and its low IL-18 : IL-1*β* ratio can also be viewed as an IL-1*β* dominant inflammation. SiO_2_ and other particle exposure are known to be correlated with an increased risk of systemic autoinflammatory diseases, cardiovascular events, and lung disease, such as COPD, which are also strongly related to IL-1*β* [53–56], and that can benefit from IL-1*β* blocking treatment [57].

Furthermore, the importance of ratios between polarizing cytokines in cell differentiation and the immunological response have been suggested by Zielinski et.al [18], but the relevance of the ratio between IL-1*β* and IL-18 have not been studied. The diverse ratios also show that the NLRP3 inflammasome activity is not static and can give a dynamic response, depending on the inducers. This flexibility of the NLRP3 inflammasome has previously been implicated by Schmidt and Lenz [58] and Bezbradica et al. [59], who demonstrate that the NLRP3 inflammasome show a greater response when stimulated with exogenous PAMPs than endogenous DAMPs. In the current study, several attempts were made to elucidate a mechanism that could explain the difference in IL-18 : IL-1*β* ratio for SiO_2_, but no sufficient models were found. Nonetheless, differential regulation of *IL1B* and *IL18* mRNA expressions could be excluded as a mechanism, since the inducers did not affect the mRNA expression. Only LPS affected the *IL1B* expression, with a 100-fold increase compared to untreated controls, while *IL18* was constitutively expressed, as shown previously by Puren et al. [60] and Zhu and Kanneganti [61]. Likewise, mRNA expression of the inflammasome components also failed to explain why SiO_2_ gives a diverse ratio, as both chitosan and SiO_2_ show a lower PYCARD mRNA expression and chitosan alone affect the *CASP1* expression. Besides *NLRP3*, *PYCARD*, and *CASP1*, the mRNA expression of *CASP8* was also examined as caspase-8 has been previously shown to assist in inflammasome activation [48, 62] but its role here cannot be confirmed.

Additionally to IL-1*β* and IL-18, the release of other cytokines, not directly regulated by the inflammasome, were measured and are summarized in supplementary table I. Stimulation with the inducers resulted in an increased release of several cytokines compared to primed control, of which chitosan significantly affected IL-6 and TNF compared to ATP and SiO_2_ stimulation. The released IL-6 and TNF is likely a secondary response to the inducers, mediated indirectly by IL-1*β* or IL-18 as the release did not occur in the THP-1 knockout cells deficient of NLRP3 and caspase-1 (supplementary figure 3). However, this secondary response could not clearly be correlated to the IL-18 : IL-1*β* ratio. Furthermore, the difference in IL-18 : IL-1*β* ratio could not be attributed to the caspase-1 activity as chitosan and SiO_2_, but not ATP, showed a higher percentage of cells positive for FLICA 24 h after stimulation, when compared to LPS-primed controls. A difference in time kinetics between the three inducers may lead to a bias in FLICA-positive cells at 24 h. However, the ratios described in this paper are derived from the accumulated production and release of inflammasome-activated cytokines at 24 h in order to dilute the effect of inflammasome time kinetics. Next, as induction of cell death and the subsequent unspecific membrane leakage may affect the amounts of cytokines available for extracellular detection, extracellular LDH was used to measure cell lysis. ATP induced the release of IL-1*β* without an increase of cell lysis compared to the controls. It is therefore likely that ATP induces GSDMD pores while escaping a pyroptotic fate, which have been demonstrated as a possible alternative to pyroptosis [48, 63]. Furthermore, the percentage of lysed cells did not reach over 30% after stimulation with chitosan and SiO_2_ which means that the majority of the cells are still intact after 24 h. However, since chitosan and SiO_2_ both increased the percentage of lysed cells, the difference in the IL-18 : IL-1*β* ratio cannot be explained by pyroptosis.

## 5. Conclusions

Taken together, our data suggest that the different inflammasome inducers lead to a diverse functional outcome that goes beyond the direct production of the hallmark cytokine IL-1*β*, irrespective of gene expression, cell lysis, and caspase-1 activity. The differential regulation of IL-1*β* and IL-18 cleavage in human cells need further elucidation, and given the fact that the NLRP3 inflammasome reacts to such a vast array of molecules and have been implicated in numerous, clinically distant diseases, a more holistic approach to studying inflammasome activation under different conditions is warranted. In conclusion, this study shows that the NLRP3 inflammasome is capable of directly tailoring the specific response to a particular stimulus. By taking into account the ratio between IL-1*β* and IL-18, it is possible to increase our understanding of how different inflammasome inducers affect inflammasome functionality and thus the subsequent immune responses.

## Data Availability

The data used and analyzed in this paper can be obtained from the corresponding authors with reasonable requests.

## Supplementary Materials

Supplementary Materials Supplementary Figure 1. Different doses of LPS affects the release of inflammasome cytokines. The concentration of released IL-1*β* and IL-18 were detected 24 h after stimulation of THP-1 cells with ATP, chitosan, and SiO_2_. Cells were primed with (a) 1 *μ*g/mL or (b) 100 ng/mL LPS prior to stimulation. The concentration cytokines were normalized against the mean of the control (primed with LPS). Data are shown as mean ± SD from six individual experiments. Supplementary Figure 2. Dose response of the inflammasome inducers ATP, chitosan, and SiO_2_. Undifferentiated THP-1 cells were primed with LPS and stimulated with increasing doses of (a) ATP (mM), (b) chitosan (*μ*g/mL), or (c) SiO_2_ (*μ*g/mL). Isolated primary monocytes were primed with LPS and stimulated with increasing doses of (d) ATP (M), (e) chitosan (g/mL), or (f) SiO_2_ (g/mL). The concentration of released IL-1*β* was measured 24 h after stimulation. The selected doses are marked with arrows. Data are shown as mean ± SD from three individual experiments. Supplementary Figure 3. Lack of inflammasome components affect inflammasome-independent cytokines. THP-1 monocytes with functional NLRP3 inflammasome (wt) and inflammasome-deficient THP-1 cells, lacking either functional NLRP3, ASC, or caspase-1 proteins, were primed with LPS and stimulated with ATP, chitosan, or SiO_2_ for 24 h. Controls were treated with LPS alone. The concentration of released (a) IL-6, and (c) TNF were measured. The relative expression of (b) IL-6, and (d) TNF shown is compared to reference genes TBP and HPRT1. Data are shown as mean ± SD from six individual experiments. Supplementary Table I. Release of noninflammasome-related cytokine and chemokines. Human inflammatory panel 1 (BioLegend), a multiplex bead-based flow cytometry detection, was used according to the manufacturer's instructions to detect the concentrations of selected cytokines and chemokines from LPS-primed THP-1 cells following stimulation with ATP, chitosan, or SiO_2_ for 24 h. Some cytokines could not be detected (ND) within the range of the kit. Data are shown as mean from six individual experiments.
