# Supplementary material for: Molecularly Distinct NLRP3 Inducers Mediate Diverse Ratios of Interleukin-1β and Interleukin-18 from Human Monocytes
Source: Mediators Inflamm. 2020 Oct 22;2020:4651090. doi: 10.1155/2020/4651090 (PMC7599400; doi:10.1155/2020/4651090)
Supplement: Supplementary Materials — Supplementary Figure 1. Different doses of LPS affects the release of inflammasome cytokines. The concentration of released IL-1β and IL-18 were detected 24 h after stimulation of THP-1 cells with ATP, chitosan, and SiO2. Cells were primed with (a) 1 μg/mL or (b) 100 ng/mL LPS prior to stimulation. The concentration cytokines were normalized against the mean of the control (primed with LPS). Data are shown as mean ± SD from six individual experiments. Supplementary Figure 2. Dose response of the inflammasome inducers ATP, chitosan, and SiO2. Undifferentiated THP-1 cells were primed with LPS and stimulated with increasing doses of (a) ATP (mM), (b) chitosan (μg/mL), or (c) SiO2 (μg/mL). Isolated primary monocytes were primed with LPS and stimulated with increasing doses of (d) ATP (M), (e) chitosan (g/mL), or (f) SiO2 (g/mL). The concentration of released IL-1β was measured 24 h after stimulation. The selected doses are marked with arrows. Data are shown as mean ± SD from three individual experiments. Supplementary Figure 3. Lack of inflammasome components affect inflammasome-independent cytokines. THP-1 monocytes with functional NLRP3 inflammasome (wt) and inflammasome-deficient THP-1 cells, lacking either functional NLRP3, ASC, or caspase-1 proteins, were primed with LPS and stimulated with ATP, chitosan, or SiO2 for 24 h. Controls were treated with LPS alone. The concentration of released (a) IL-6, and (c) TNF were measured. The relative expression of (b) IL-6, and (d) TNF shown is compared to reference genes TBP and HPRT1. Data are shown as mean ± SD from six individual experiments. Supplementary Table I. Release of noninflammasome-related cytokine and chemokines. Human inflammatory panel 1 (BioLegend), a multiplex bead-based flow cytometry detection, was used according to the manufacturer's instructions to detect the concentrations of selected cytokines and chemokines from LPS-primed THP-1 cells following stimulation with ATP, chitosan, or SiO2 for 24 h. Some cy [file 4651090.f1.pdf]

Supplementary Materials

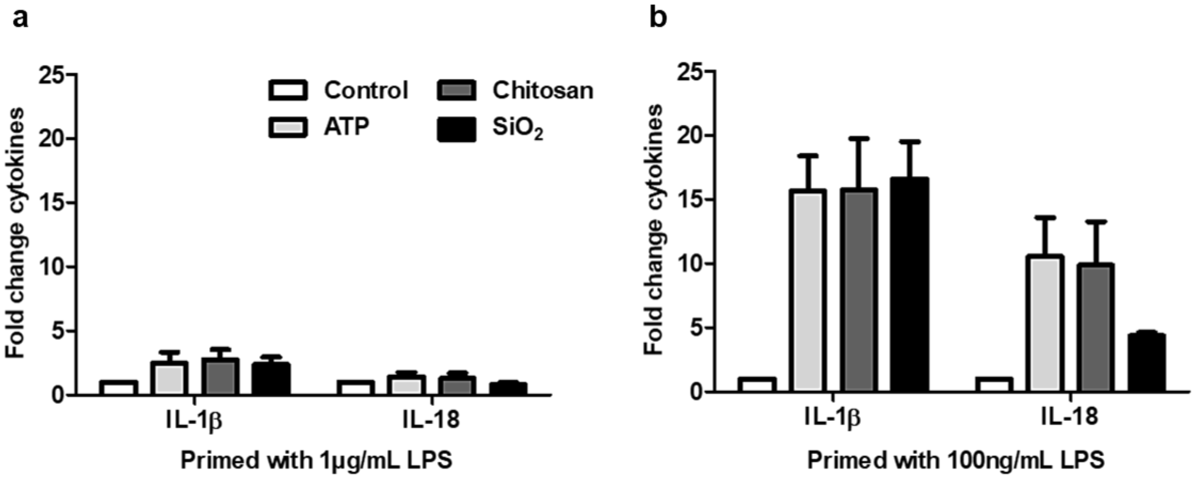

Supplementary Figure 1. Different doses of LPS affects the release of inflammasome cytokines. The concentration of released IL-1β and IL-18 were detected 24 h after stimulation of THP-1 cells with ATP, chitosan and SiO_2. Cells were primed with (a) 1 µg/mL or (b) 100 ng/mL LPS prior to stimulation. The concentration cytokines were normalized against the mean of the control (primed with LPS). Data are shown as mean +/- SD from six individual experiments.

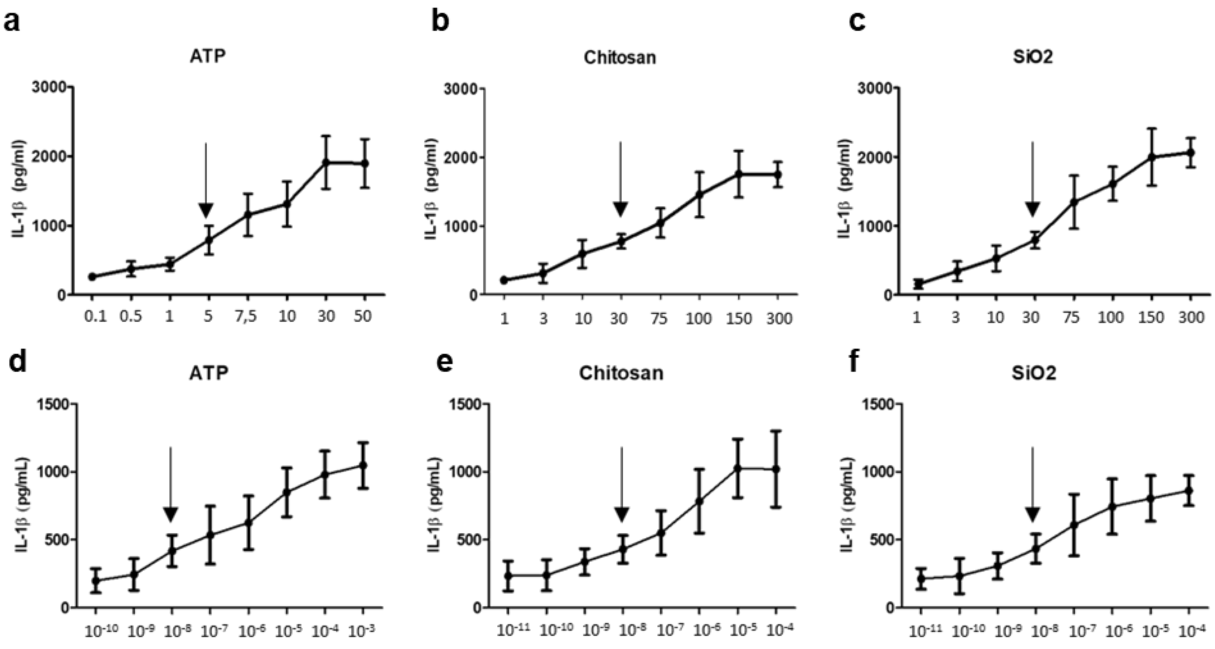

Supplementary figure 2. Dose-response of the inflammasome inducers ATP, chitosan and SiO_2. Undifferentiated THP-1 cells were primed with LPS and stimulated with increasing doses of (a) ATP (mM), (b) chitosan (µg/mL) or (c) SiO_2 (µg/mL). Isolated primary monocytes were primed with LPS and stimulated with increasing doses of (d) ATP (M), (e) chitosan (g/mL) or (f) SiO_2 (g/mL). The concentration of released IL-1β were measured 24h after stimulation. The selected doses are marked with arrows. Data are shown as mean +/- SD from three individual experiments.

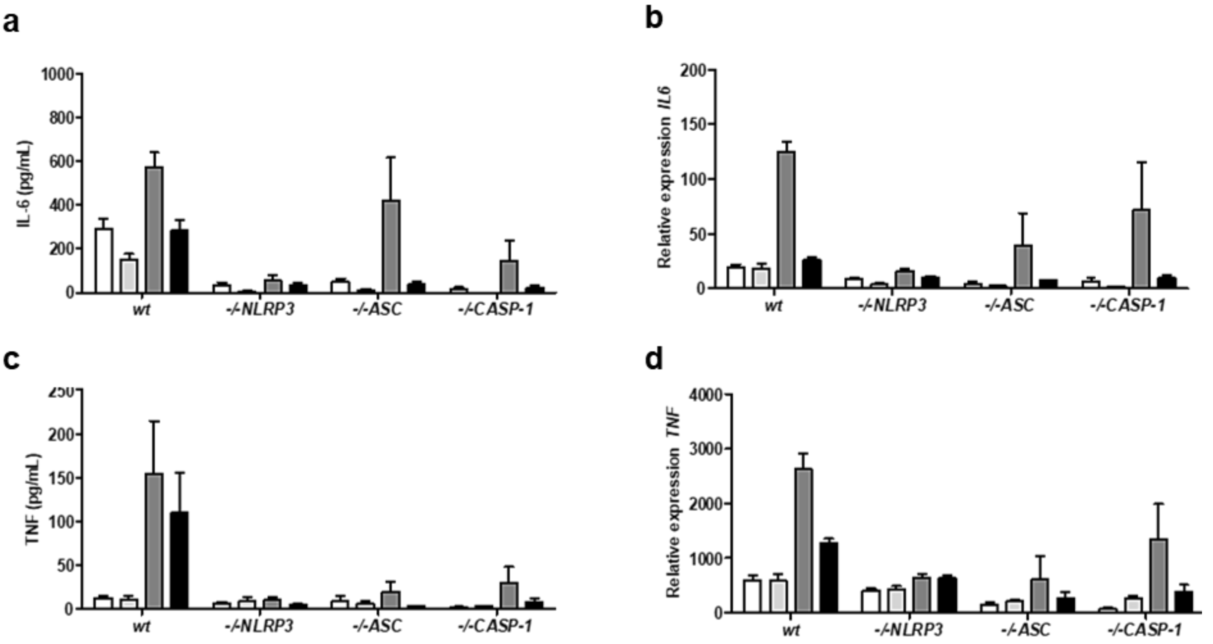

Supplementary figure 3. Lack of inflammasome components affect inflammasome-independent cytokines. THP-1 monocytes with functional NLRP3 inflammasome (wt) and inflammasome deficient THP-1 cells, lacking either functional NLRP3, ASC or caspase-1 proteins, were primed with LPS and stimulated with ATP, chitosan or SiO_2 for 24 h. Controls were treated with LPS alone. The concentration of released (a) IL-6, and (c) TNF were measured. The relative expression of (b) IL-6, and (d) TNF shown are compared to reference genes TBP and HPRT1. Data are shown as mean +/- SD from six individual experiments.

Supplementary Table I Release of non-inflammasome related cytokine and chemokines. The human inflammatory panel 1 (BioLegend), a multiplex bead-based flow cytometry detection, were used according to manufacturer's instructions to detect the concentrations of selected cytokines and chemokines from LPS-primed THP-1 cells following stimulation with ATP, chitosan or SiO2 for 24h. Some cytokines could not be detected (ND) within the range of the kit. Data (in pg/mL) are shown as mean from six individual experiments.

	Neg. control	Control	ATP	Chitosan	SiO_2
CCL-2	54	622	4 433	7 166	7 846
CXCL-8	5	549	5 712	5 517	5 357
IFN-A	ND	ND	ND	ND	ND
IFN- γ	ND	ND	ND	ND	ND
IL-6	ND	293	148	570	285
IL-10	ND	8	2	21	24
IL-12	ND	ND	ND	ND	3
IL-17	ND	3	3	5	6
IL-23	8	62	68	68	96
IL-33	ND	27	29	58	50
TNF	1	12	11	153	110
